# Can dogs help chickens? Pet owners’ willingness to pay for animal welfare-friendly pet food in the United States

**DOI:** 10.1017/awf.2022.3

**Published:** 2023-01-30

**Authors:** Hillary Pearce, Clinton L Neill, Kenneth Royal, Monique Pairis-Garcia

**Affiliations:** 1 London SW19 1BW, UK; 2 Department of Population Medicine and Diagnostic Sciences, Cornell University College of Veterinary Medicine, Ithaca, NY 14853, USA; 3 Office of Academic Affairs, Virginia-Maryland College of Veterinary Medicine, Blacksburg VA 24061, USA; 4 Department of Population Health and Pathobiology, North Carolina State University College of Veterinary Medicine, Raleigh, NC 27606, USA

**Keywords:** animal welfare, animal welfare marketing, farm animal welfare, pet food, pets, willingness-to-pay

## Abstract

Consumer concern about farmed animal welfare is growing but does not always translate into real-world purchasing behaviour of welfare-friendly animal products for human consumption. Possible reasons for this include unfamiliarity with farming practices and economic sensitivity. In contrast, the number and role of pets in the United States have grown measurably, and spending on pets is strong. The pet food market has many opportunity niches as pet owners navigate strong marketing trends and nutrition philosophies. We hypothesised that pet owners in the US would be willing to pay a premium for pet food containing welfare-friendly animal ingredients. Eight hundred and thirty-eight pet owners completed an online survey asking questions that measured their knowledge of and interest in farm animal welfare, and their willingness-to-pay for pet food labelled as farm animal welfare-friendly. Respondents overall displayed relatively low knowledge about farm animal welfare, but poor self-assessment of their own knowledge. They displayed interest in farm animal welfare and an overall positive mean willingness-to-pay (WTP) for welfare-friendly pet food. Younger respondents, women and cat owners displayed a higher WTP than older respondents, men and dog owners. Income level was not correlated to WTP. Creating pet food products that contain animal ingredients produced using welfare-friendly practices may enhance farm animal welfare via two primary avenues: by providing a sustainable and value-added outlet for the by-products of welfare-friendly human food products, and by providing an educational opportunity about farm animal production via pet food packaging and other advertising.

## Introduction

### Pets and pet food

The roles that animals play in human society have markedly evolved throughout history. Following our earliest interactions with wild animals as predators to be avoided and prey to be hunted, humans first domesticated wolves, likely as hunting aids. In the last 200 years, our relationship with animals has evolved from utilising them purely as resources to include companionship (Rivto 1987). In the United States, it is increasingly common for pets to be treated and described as members of the family (Boya *et al.* 2012) or even as extensions of their owners’ sense of self (Jyrinki & Leipämaa-Leskinen 2006). In 2020 alone, nearly 73.5 million American households owned a pet and $US103.6 billion was spent caring for these animals (Packaged Facts 2020). Even in the face of adversity the pet industry continues to grow and spending on pet food remains a constant (Henderson 2013). In a survey conducted in 2020 (the first year of the COVID-19 pandemic), 61% of respondents reported an overall concern regarding finances, but pet-related spending was not impacted (American Pet Products Association [APPA] 2020b). In fact, an index of pet industry stocks outperformed the S&P500 by 48%, suggesting priority of pet care was not influenced (Wall 2021).

Pet food is a $US42 billion industry in America and is expected to grow even larger (APPA 2020a). Premium and niche products garner high prices, often mimicking human food trends, such as gluten/grain-free, certified organic, exotic proteins and raw food, and therapeutic foods designed to help manage specific illnesses.

In addition to perceived nutritional quality, sustainability is a consideration for some consumers. Meeker and Meisinger (2015) define sustainability as it relates to pet food as “the ability to produce pet food that provides sufficient energy and the amounts of essential nutrients required to maintain good health now and into the future with the smallest possible environmental foot-print.” In the US, most commercial pet foods utilise by-products from the human food system. The Association of American Feed Control Officials defines ‘by-product’ as it relates to pet food as “secondary products produced in addition to the principal product” (Association of American Feed Control Officials [AAFCO] 2021). What people prefer as principal food differs by geographic region, culture, and over time. Approximately 25 million tons of animal by-products are rendered in the US each year (Meeker & Meisinger 2015). These animal products are as safe and nutritious as the primary skeletal meat cuts commonly sold for human consumption but would otherwise go to waste.

### Farm animal welfare

A recent and related area of concern for consumers is the welfare of food animals. Although most consumers are relatively ill-informed regarding modern animal production (Heinke & Theuvsen 2017; Stampa *et al.* 2020), they have more access to information about animal welfare than ever before thanks to the internet and global media (Toma *et al.* 2010; Tonsor & Olynk 2011; McKendree *et al.* 2014). This rising concern is often measured in terms of willingness-to-pay (WTP) (Napolitano *et al.* 2010; Nocella *et al.* 2010; Lusk & Norwood 2011).

In their review, Stampa *et al.* (2020) found that a variety of consumer segments were interested in and/or willing to pay a premium for pasture-raised products. In the US, they found that younger consumers were particularly interested in these products in line with their greater concern for environmental responsibility.

### Opportunity for animal welfare-enhanced pet food

There is limited literature on the relationship between the deepening pet-owner bond and increasing public interest in farm animal welfare. In a Dutch study, pet owners perceived farm animal quality of life lower than did non-pet owners (Boogaard *et al.* 2006). In the US, despite a large pet-owning population, the relatively small market for animal welfare-enhanced products implies they are primarily purchasing conventionally raised food, regardless of any declared concern or WTP. They purchase agricultural products raised using practices that would be considered unacceptable for companion animals.

It is also possible that pet owners are completely abstaining from purchasing animal-based products for their own consumption. Since the vast majority of commercial pet foods contain meat, these individuals face what Rothgerber (2013) referred to as The Vegetarian’s Dilemma. Feeding companion animals a diet that conforms to the owner’s avoidance of meat products may jeopardise the pet’s welfare. This is particularly true for cats, who are obligate carnivores. In their study on the prevalence of plant-based diets for pets, Dodd *et al.* (2019) found that vegetarians/vegans were over-represented among pet owners compared to the general population. Nearly half of the vegan respondents said they wished to feed their pets a plant-based diet but did not because of nutritional concerns.

This begs the question as to whether pet food containing animal welfare-enhanced ingredients would find a sizable market niche. Consumers appear to focus on purchasing healthy food for their dogs even more than for themselves, and price sensitivity is lower for pet food than for groceries (Tesform & Birch 2010; Boya *et al.* 2012). Ethical vegetarians/vegans were more likely to own pets than those who were health-focused, and also felt more guilty about feeding meat-based diets to their pets (Rothgerber 2013). One German study thoroughly discussed these connections between companion animal ownership and WTP for animal welfare-enhanced foods for human consumption (Pirsich *et al.* 2017) but, to the authors’ knowledge, no studies have investigated whether pet owners would be willing to pay for animal welfare-enhanced pet food.

Therefore, the aim of this study was to survey pet owners in the US about their perceived and actual knowledge of farm animal welfare, their interest in farm animal welfare, and their WTP for pet food labelled as containing animal-welfare enhanced ingredients.

## Materials and methods

### Ethical approval

The survey protocol was approved and deemed exempt from the policy as outlined in the Code of Federal Regulations (Exemption: 46.101. Exempt d2) by the North Carolina State University Institutional Review Board, Protocol #23465. Survey participants were recruited via Amazon’s mTurk platform, a crowdsourcing marketplace in which ‘workers’ are compensated a nominal fee to complete surveys. Numerous studies have shown samples obtained from mTurk are more socioeconomically and culturally diverse than samples typically obtained from other means (Casler *et al.* 2013). Further, the sample frame consisted of participants located throughout the United States, as opposed to a limited geographic area. All participants were required to electronically sign an informed consent form to participate in the survey. Each respondent was aware of the purpose of the survey, that the data would be used for research purposes, and that their responses could not be traced back to them to ensure anonymity.

### Survey design

**Figure 1.**
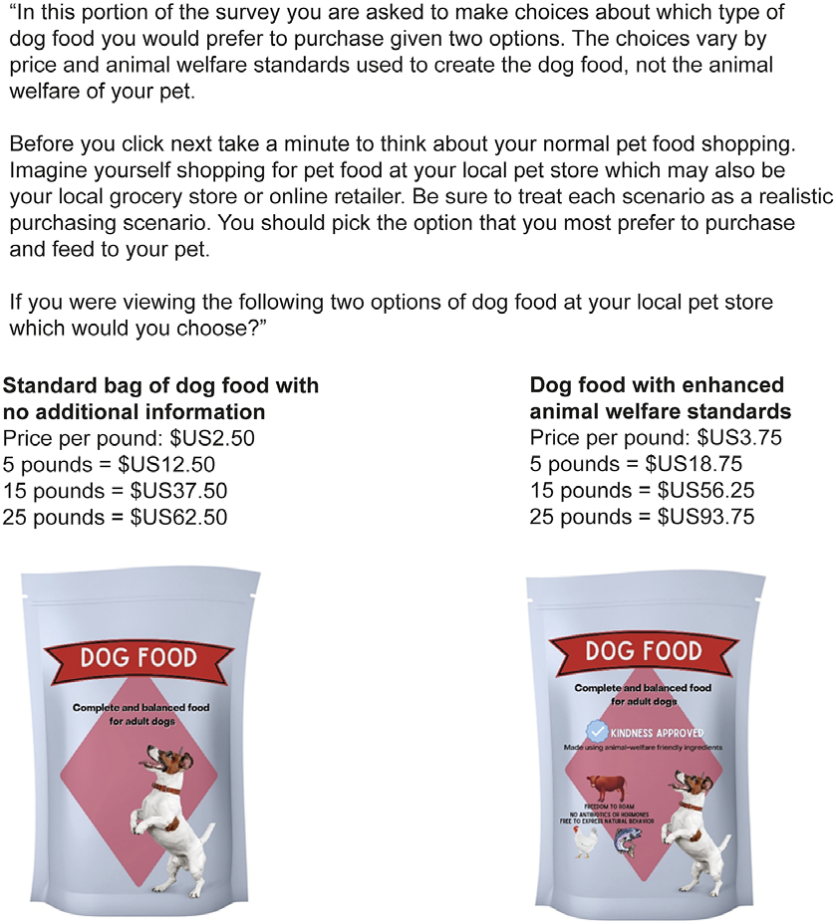
Example of the dichotomous choice contingent valuation question and images presented to dog owner survey participants.

The survey was distributed online via Amazon’s mTurk platform between November 30 and December 1, 2020. The survey was structured in three parts. Part 1 consisted of demographic questions including pet ownership and responsibility for household purchasing decisions (e.g. groceries and pet food). Part 2 comprised nine Likert-scale questions designed to assess participants’ knowledge level and attitudes about farm animal welfare in the US (Table 1). Question 1 gauged the participants’ self-assessed level of knowledge regarding farm animal welfare. Questions 2 and 3 gauged participants’ actual level of knowledge by presenting them with commonly held misconceptions regarding animal agriculture in America. Questions 4–9 measured participants’ interest in farm animal welfare, including their perception of their influence via purchasing behaviour. Part 3 assessed participants’ WTP for dog or cat food labelled with indicators that the livestock used as ingredients experienced enhanced welfare. The welfare-enhanced dog and food images shown to participants contained a fictitious ‘Kindness-approved’ label, and the statements ‘Made using animal welfare-friendly ingredients’, ‘Freedom to roam’, ‘No antibiotics or hormones’, and ‘Freedom to express natural behaviour’ alongside graphics of a cow, a chicken, and a fish. The reference and welfare-enhanced pet food images can be seen in Figure 1. All participants were permitted to complete the demographic and farm animal welfare parts of the survey. Inclusion criteria to advance to the WTP section included being a cat and/or dog owner and being responsible for ≥ 50% of household purchasing decisions.

**Table 1.** Survey questions answered by respondents using a five-point Likert scale (strongly disagree, somewhat disagree, neither agree nor disagree, somewhat agree, strongly agree)

Question	
1	I am knowledgeable about the conditions in which animals (e.g. pigs, meat and egg-laying chickens, dairy and beef cows) are kept in US agriculture
2	Animals raised for food in the US typically have the ability to freely stand up, lie down, turn around and (if applicable) fully extend their wings
3	Pigs and chickens raised for meat in the US typically have access to the outdoors for at least part of the day
4	It is important to meet the behavioural and social needs of farm animals in addition to keeping them physically healthy
5	When doing grocery shopping, I think about animal welfare
6	I would like to have more information about livestock farming when purchasing meat
7	I am interested in the living conditions of the animals that provide the meat I purchase
8	In the US sufficient attention is paid to animal welfare in livestock farming
9	Through my buying behaviour, I have an influence on how animals are raised in agriculture

The expansive literature on economic derivations of WTP presents several options on incentive-compatible designs. The two most common designs are choice-based conjoint designs (also referred to as choice experiments) and contingent valuation (CV) methods. For this study, we employed a double-bounded dichotomous choice CV approach for two reasons: (i) brevity of questions in a larger survey, which reduces survey fatigue; and (ii) to analyse the direct effects of demographic factors on WTP (Carson 1985; Hanemann 1985). Choice experiments often require a larger number of questions and do not allow demographic factors to enter the model except via interaction terms. Also, a double-bounded dichotomous choice CV is still incentive-compatible like a choice experiment (Carson & Hanemann 2005). This part of the survey was performed as follows:

- (1) The participant was presented with a reference pet food that did not have any animal welfare information on the packaging and with an alternative product that included indication of enhanced animal welfare standards on the packaging. The reference pet food had a price that remained constant throughout the survey. Participants were randomly assigned a scenario in which the price for the welfare-enhanced pet food could be greater than, less than, or equal to the reference pet food.
- (2a) If the participant chose the welfare-enhanced pet food, they were presented with the same scenario again, but the price of the welfare-enhanced option was increased.
- (2b) If the participant chose the reference food, they were presented with the same scenario again, but the price of the welfare-enhanced option was decreased.

This type of design creates bounds on a participant’s WTP for welfare-enhanced pet food and is useful when constructing market demand estimations.

Participants indicated what type of pet they owned; dog, cat, and/or other. If the participant was only a dog or cat owner, they were presented with scenarios pertaining to that pet type. If the participant indicated they had both a dog and cat in the home, they were randomly assigned to either the dog or the cat food version of the survey. If the participant indicated that they have neither a dog nor a cat, but some other type of pet, then they did not participate in the CV portion of the survey. The prices used in the survey were reflective of the current marketplace, as determined by the 2020 Woof Whiskers Cost of Dog Food Study (published 2021) and direct pricing research on Chewy.com, a common pet food online retailer. The average (and reference) prices of cat and dog foods were $US3.00 and $US2.50 per pound, respectively. In all scenarios, prices were presented both on a per pound basis and total cost for common bag sizes (5, 15, and 25 pounds for dogs; 5 and 12 pounds for cats). For an example of the initial scenario, see Figure 1.

### Statistical analysis

Contingent valuation data are typically analysed via censored regressions. Censored regressions are similar to linear regressions except the dependent variable is bounded between two points rather than one distinct point estimate. Moreover, the regression model is estimated using maximum likelihood methods rather than ordinary least squares. Due to the bounded/censored form of the dependent variable, the likelihood function for the model is formulated as follows (Neill & Williams 2016; Neill & Holcomb 2019):
(1)

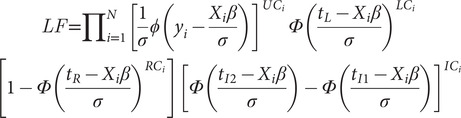

where 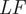 is the likelihood function for each cluster; 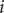 is the individual within the cluster; 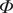 is the cumulative standard normal distribution function and 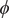 is the standard normal probability density function; 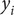 is the observed WTP value for individual *i*; *X* represents a matrix of demographic variables and 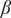 is a vector of coefficients; 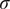 is the standard deviation of the error term; 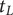 , 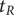 , 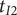 , 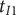 are the left point of censorship, right point of censorship, upper (second) point of censorship in interval censor, and the lower (first) point of censorship in interval censor, respectively; 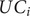 , 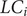 , 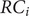 , 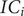 are indicator variables representing uncensored, left censored, right censored, and interval censored observations, respectively.

In the case of this study, the censored regression model is performed for the dog and cat responses separately given the different price ranges. The linear representation of the model with demographic factors as explanatory variables can be written as:
(2)

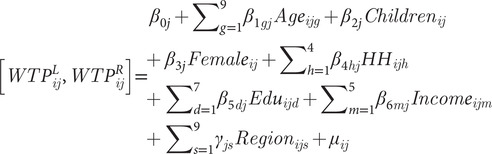

where 
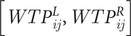
 is the left censored, *L*, and right censored, *R*, WTP dependent variable for respondent *i* for pet type *j*; *Age* is a categorical variable for age range with 18–23 years of age as the reference group; *Children* is an indicator variable for whether there are children under the age of 18 living in the household; *Female* is an indicator variables for those respondents that self-identified as such; 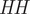 is a categorical variable that represents the number of people currently living in the household with one being the reference category; *Edu* is a categorical variable for the level of education the participant has with ‘four-year degree’ as the reference category; *Income* is a categorical variable for the amount of annual household income before taxes with ‘Less than $US30,000’ as the reference category; and 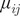 is the normally distributed regression error. Each of the demographic variables are included to align with standard marketing and economics literature (for examples, see Lusk & Norwood 2011; Neill & Williams 2016; Neill & Holcomb 2019).

From the Likert-scale questions assessing participants’ knowledge and attitudes towards farm animal welfare, two different measures are constructed, and the means are compared across different demographic groups within the sample. The first measure is a *Knowledge difference* score which is the difference between the self-assessed and actual knowledge Likert-scale questions. First, the second and third Likert-scale question responses are averaged to obtain the actual knowledge score. Second, the self-assessed knowledge Likert-scale response is subtracted from the composite actual knowledge score. So, the *Knowledge difference* measure is the difference between actual and self-assessed knowledge. A positive value to this measure indicates that participants are more knowledgeable than they think about animal welfare, while a negative value would indicate that they are less knowledgeable than they think. A zero would indicate that they are perfectly accurate in their knowledge assessment.

The second measure constructed from the remaining Likert-scale questions is an *Interest score* which indicates how interested a participant is in farm animal welfare and products with welfare enhancements. This score is the addition of all Likert-scale scores from Questions 4–9 in Table 1. The possible values range from six to 30. A low *Interest* score would range from six to 13, a medium score would range from 14 to 21, and a high score would range from 22 to 30. Again, both measures constructed from the Likert-scale questions are compared across demographic characteristics (age, education, income, gender, and pet ownership) by comparing mean values and 95% confidence intervals.

## Results

Eight hundred and thirty-eight participants completed the survey resulting in a margin-of-error of 3.39%. All 838 were dog and/or cat owners. Of these, 250 completed cat food scenarios and 588 completed dog food scenarios. The results of the censored regression for each pet type are presented in Table 2. As mentioned in the previous section, the independent/explanatory variables are predominantly binary or categorical and must be interpreted in reference to a base alternative. The coefficient values also represent the marginal WTP for the respective animal welfare-enhanced pet food. For example, female dog and cat owners had a higher marginal WTP for animal welfare-enhanced pet food (as compared to a pet food with no welfare information) on the order of +$US0.18 and +$US0.35 per pound, respectively, relative to male dog and cat owners. Conversely, the presence of children in the household decreased WTP for animal welfare-enhanced pet food by $US0.35 and $US0.42 for dog and cat owners, respectively. The *BID* coefficient is a theoretical check to determine if the percentage of participants who are presented with a higher bid accept said bid. In other words, if a participant is presented with a higher bid, they should have a decreased likelihood to accept that bid. Thus, a negative value is theoretically consistent.

**Table 2.** Coefficient estimates from censored regressions on WTP for animal welfare-enhanced pet food

	Dog (n = 589)		Cat (n = 250)	
Coefficients	Estimate	SE	Estimate	SE
Intercept	2.82***	0.35	2.07***	0.50
Female	0.18*	0.10	0.35*	0.16
Children in the household	–0.35***	0.12	–0.42**	0.18
*Age (reference = 18-23)*				
24–29	–0.50*	0.30	–0.17	0.39
30–35	–0.21	0.30	–0.02	0.39
36–40	–0.37	0.34	0.36	0.42
41–45	–0.77**	0.34	–0.48	0.45
46–50	–0.68**	0.34	0.52	0.50
51–55	–0.84**	0.34	0.00	0.50
56–60	–0.73*	0.38	–0.37	0.48
61–65	–0.40	0.44	0.12	0.55
Older than 65	0.13	0.53	–0.19	0.83
*Household size (reference = 1)*				
Two	0.03	0.16	0.63**	0.27
Three	0.06	0.16	0.33	0.27
Four	0.26*	0.15	0.31	0.29
Five or more	0.22	0.23	0.53	0.36
*Education (reference = four-year Degree)*				
Less than High School	–1.01	0.88	0.06	0.71
High School/GED	0.26	0.22	0.15	0.32
Some college	0.34	0.21	0.11	0.24
2-year degree	0.28	0.22	–0.23	0.27
Master’s degree	–0.39***	0.12	–0.06	0.22
Doctorate degree	–0.61	0.48	–0.40	0.71
Professional degree (JD, MD, DVM)	0.78*	0.43	.	.
*Income (reference = less than $US30,000)*				
$US30,000 – $US59,999	–0.10	0.14	0.57**	0.24
$US60,000 – $US89,999	–0.11	0.15	0.34	0.25
$US90,000 – $US119,999	0.31*	0.18	0.30	0.29
$US120,000 – $US149,999	0.38	0.30	0.13	0.44
$US150,000 or more	5.67	67.4	0.15	0.86
BID	–0.63***	0.03	–0.65***	0.05
Log-likelihood:	–705.33		–311.72	

*, **, and *** indicate statistical significance at the 10%, 5%, and 1% levels, respectively.

WTP: Willingness-to-pay.

There was much heterogeneity within categorical demographic variables. For dog owners, increases in age generally decreased WTP with varying magnitudes and statistical significance across age categories, holding all else equal. Dog owners aged 51–55 years had the lowest WTP (–$US0.84 compared to 18–23-year-old dog owners). In fact, since there was no age category with a statistically significant positive coefficient, it can be said that 18–23 year old dog owners had the highest WTP. For household size, only households with four members showed slight statistical significance (*P* = 0.10) and had a higher WTP (+$US0.24) than dog-owning households with only one member. Dog owners with a Masters degree had a lower WTP (–$US0.39), and those with a Professional degree had a higher WTP (+$US0.78), than those with a four-year degree. Within income categories, only households that make $US90,000 to $US119,999 had a slightly statistically different (*P* = 0.10) WTP (+$US0.31) compared to those making less than $US30,000 a year.

For cat owners, age and education did not statistically affect WTP for animal welfare-enhanced cat food. In fact, the only categorical variables that impacted WTP statistically were households with two members (marginal WTP = +$US0.63) as compared to one, and households with incomes of $US30,000 to $US59,999 (marginal WTP = +$US0.57) as compared to households with incomes less than $US30,000.

Using the coefficient values from Table 2 and average values from Tables 3 and 4, demand curves for the average dog and cat owner were simulated. It is important to note the survey population was national in scope, which has been a limiting factor in previous studies. From the demographics and summary statistics in Tables 3 and 4, we find that our sample had slightly more males and higher education levels than the national statistics. In addition, our sample was relatively similar to the national average on income and household size. Overall, our sample is relatively similar to the national average and of sufficient sample size to have a small margin-of-error. Moreover, while the sample is not perfectly representative of the US, the sample does capture a diverse set of pet owners which is key to answering the market viability question of animal welfare-enhanced pet food.

**Table 3.** Summary statistics for dog owners that participated in the animal welfare-enhanced dog food WTP Experiment (n = 589)

	Percentage	SD
Female	39.22%	0.48
Children in the household	70.80%	0.45
Also a cat owner	21.73%	0.41
*Age*		
18–23	4.41%	0.20
24–29	28.01%	0.44
30–35	29.37%	0.45
36–40	8.49%	0.27
41–45	7.47%	0.26
46–50	7.47%	0.26
51–55	6.62%	0.24
56–60	4.07%	0.19
61–65	2.21%	0.14
Older than 65	1.87%	0.13
*Household size*		
One	15.96%	0.36
Two	23.09%	0.42
Three	23.09%	0.42
Four	30.90%	0.46
Five or more	6.96%	0.25
*Education*		
Less than High School	0.34%	0.05
High School/GED	5.26%	0.22
Some college	7.64%	0.26
2-year degree	5.43%	0.22
4-Year degree	56.54%	0.49
Master’s degree	22.07%	0.41
Doctorate degree	1.02%	0.10
Professional degree (JD, MD, DVM)	1.70%	0.12
*Income level*		
Less than $US30,000	16.13%	0.36
$US30,000 – $US59,999	33.28%	0.47
$US60,000 – $US89,999	31.58%	0.46
$US90,000 – $US119,999	14.09%	0.34
$US120,000 – $US149,999	3.57%	0.18
$US150,000 or more	1.36%	0.11

WTP: Willingness-to-pay.

**Table 4.** Summary statistics for cat owners that participated in the animal welfare-enhanced cat food WTP experiment (n = 250)

	Percentage	SD
Female	42.40%	0.49
Children in the household	58.80%	0.49
Also a dog owner	50.40%	0.50
*Age*		
18–23	4.40%	0.20
24–29	24.40%	0.43
30–35	28.00%	0.44
36–40	13.60%	0.34
41–45	8.00%	0.27
46–50	6.00%	0.23
51–55	5.20%	0.22
56–60	5.60%	0.23
61–65	4.00%	0.19
Older than 65	0.80%	0.08
*Household size*		
One	11.60%	0.32
Two	30.40%	0.46
Three	28.80%	0.45
Four	19.60%	0.39
Five or more	9.60%	0.29
*Education*		
Less than High School	1.20%	0.10
High School/GED	8.00%	0.27
Some college	12.40%	0.33
2-year degree	10.40%	0.30
4-Year degree	50.40%	0.50
Master’s degree	16.40%	0.37
Doctorate degree	1.20%	0.10
Professional degree (JD, MD, DVM)	.	.
*Income level*		
Less than $US30,000	16.80%	0.37
$US30,000 – $US59,999	36.40%	0.48
$US60,000 – $US89,999	28.80%	0.45
$US90,000 – $US119,999	13.60%	0.34
$US120,000 – $US149,999	3.60%	0.18
$US150,000 or more	0.80%	0.08

WTP: Willingness-to-pay.

**Figure 2.**
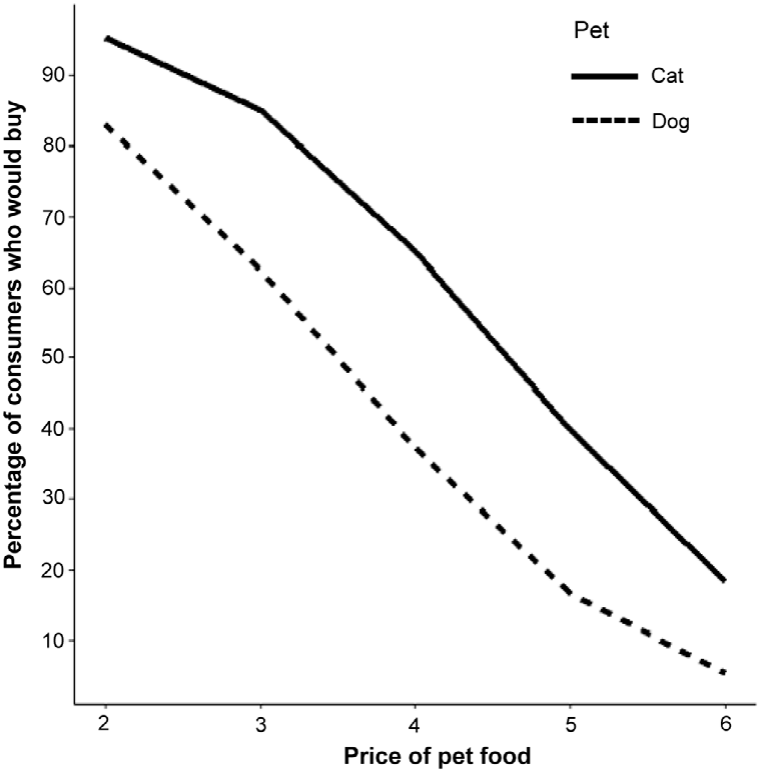
Demand curves for the sample average consumer simulated from censored regressions for animal welfare-enhanced pet food.

**Figure 3.**
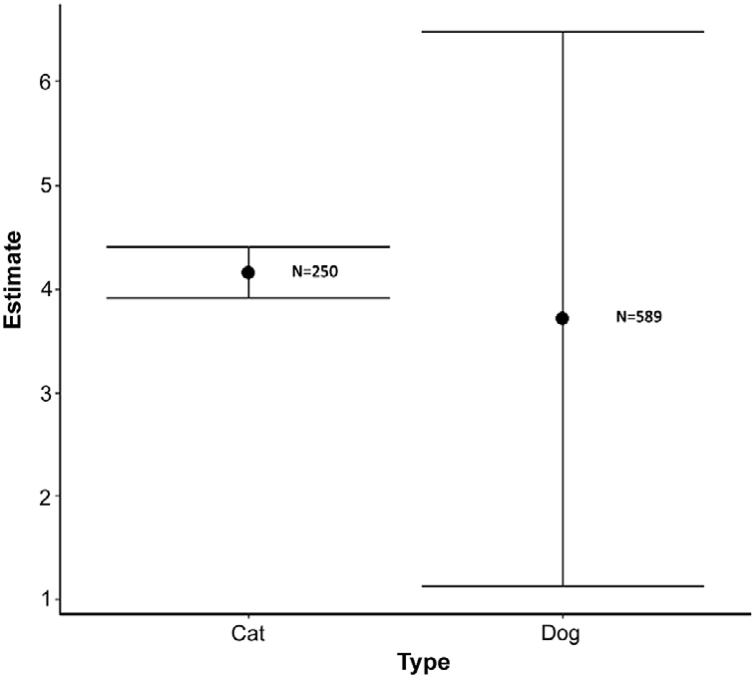
Mean willingness-to-pay for animal welfare-enhanced pet food by pet type.

As seen in Figure 2, the demand curves are theoretically consistent as fewer consumers are predicted to purchase the animal welfare-enhanced pet food as the price (bid) of the pet food increases. The animal welfare-enhanced cat food demand curve is farther to the right, indicating higher demand among cat compared to dog owners. This is confirmed in Figure 3 which presents the mean WTP for each type of pet food. Cat owners displayed a mean WTP of $US4.15 per pound for animal welfare-enhanced pet food, compared with dog owners who displayed a mean WTP of $US3.71 per pound. Cat owners also had a much smaller confidence interval that did not cross the $US3.00 per lb threshold, the price of the non-welfare-enhanced reference pet food. The dog owner confidence interval is much wider and crosses the non-welfare-enhanced reference pet food price of $US2.50 per lb. The cat owners surveyed were more consistent in their preference for the animal welfare-enhanced alternative, while the dog owners were much more heterogeneous in their preferences.

The results of the *Knowledge difference* and *Interest* composite scores are presented in Table 5. Across all demographic groups, the mean *Knowledge difference* is negative. On average, this indicates that participants know less about farm animal welfare than they think they do. Within the age categories, there is little to no statistical difference between age groups. For education, lower levels of education are closer to correctly assessing their own knowledge on animal welfare, while highly educated people are worse at assessing their knowledge. Within income groups, those with higher incomes are more accurate at assessing their own knowledge about animal welfare. There is no statistical difference between genders, but among pet owner types, cat-only pet owners are better at assessing their own knowledge about animal welfare.

**Table 5.** Means of Knowledge difference and Interest scores with corresponding 95% confidence intervals

	n	Mean Knowledge difference score	95% Confidence Interval	Mean Interest score	95% Confidence Interval
*Age*					
18–23	38	−1.04	[−1.57,−0.51]	21.47	[21.47,24.32]
24–29	228	−1.59	[−1.78,−1.40]	23.06	[23.06,24.12]
30–35	255	−1.42	[−1.60,−1.23]	22.37	[22.37,23.40]
36–40	88	−0.61	[−0.95,−0.28]	19.82	[19.82,21.77]
41–45	65	−1.65	[−2.00,−1.29]	21.96	[21.96,24.19]
46–50	61	−1.05	[−1.44,−0.66]	21.43	[21.43,23.59]
51–55	54	−1.29	[−1.74,−0.82]	21.56	[21.56,24.04]
56–60	42	−1.10	[−1.55,−0.64]	19.26	[19.26,22.22]
61–65	24	−0.98	[−1.75,−0.21]	19.60	[19.60,23.98]
Older than 65	13	−0.77	[−1.54,0.01]	20.42	[20.42,23.58]
*Education*					
Less than High School	6	−0.25	[−1.83,1.33]	15.79	[15.79,23.54]
High School/GED	56	−0.52	[−0.93,−0.11]	19.33	[19.33,21.99]
Some college	80	−0.37	[−0.70,−0.03]	19.74	[19.74,21.71]
2-year degree	60	−0.53	[−0.91,−0.14]	18.64	[18.64,21.43]
4-year degree	470	−1.48	[−1.61,−1.35]	22.96	[22.96,23.68]
Master’s degree	174	−1.80	[−2.01,−1.60]	22.84	[22.84,24.08]
Doctorate degree	11	−1.86	[−2.47,−1.26]	20.89	[20.89,26.02]
Professional degree (JD, MD, DVM)	11	−1.41	[−2.54,−0.27]	21.89	[21.89,25.93]
*Income*					
Less than $US30,000	149	−1.09	[−1.37,−0.81]	21.41	[21.41,22.94]
$US30,000 – $US59,999	293	−1.31	[−1.49,−1.14]	21.76	[21.76,22.78]
$US60,000 – $US89,999	263	−1.57	[−1.74,−1.40]	23.02	[23.02,23.94]
$US90,000 – $US119,999	120	−1.13	[−1.44,−0.82]	22.16	[22.16,23.84]
$US120,000 – $US149,999	32	−1.16	[−1.72,−0.59]	20.08	[20.08,23.36]
$US150,000 or more	11	−0.50	[−1.38,0.38]	18.96	[18.96,24.67]
*Gender*					
Male	521	−1.32	[−1.45,−1.19]	22.41	[22.41,23.13]
Female	347	−1.30	[−1.47,−1.12]	22.09	[22.09,23.07]
*Pet*					
Dog	461	−1.45	[−1.69,−1.33]	22.53	[22.53,23.32]
Cat	124	−0.63	[−0.91,−0.35]	20.40	[20.40,22.05]
Both	256	−1.51	[−1.59,−1.31]	22.92	[22.92,23.90]
Min		−4.00		6.00	
Max		4.00		30.00	

When examining the *Interest* scores across demographic groups we find that most subgroups have similar mean scores that fall into the upper end of the medium and high interest categories. Within age categories, younger age groups (under 36 years old) tend to have higher *Interest* scores. However, those in the 36–40, 56–60, and 61–65 age groups have the lowest mean *Interest* scores. Within the education subgroups, higher education leads to higher *Interest* scores, with four-year degree- and Masters degree-holders having the highest mean scores. For the income sub-groups, there is little difference between the mean values. The highest income group has the lowest mean value, but also has a wide confidence interval given the low sample size. Gender, again, does not indicate any difference in *Interest* scores, but among pet owner types, cat owners have a statistically lower mean *Interest* score.

## Discussion

In this study, we surveyed consumers to gauge their interest in and knowledge of farm animal welfare. Initially, we planned to compare these results between pet owners and non-pet owners, however all of the respondents who completed the study were owners of dogs and/or cats. We also investigated pet owners’ stated WTP for pet food labelled as produced from livestock that experienced enhanced welfare conditions.

Significant bodies of literature exist regarding consumers’ WTP for animal welfare-enhanced food for their own consumption, and regarding the intensification of the pet-owner bond and growth and diversity of the pet food market. However, to our knowledge, no studies have investigated pet owners’ WTP for animal welfare-enhanced pet food. Pirsich *et al.* (2017) acknowledged the potential for a connection between pet owners and interest in farm animal welfare. They laid interesting groundwork by surveying German grocery consumers to elucidate attitudes toward farm animal welfare of pet owners and non-pet owners, but their WTP inquiry was focused on food for human consumption rather than pet food. Their findings that pet owners were more critical of farm animal living conditions than non-pet-owners, and that pet owners were more willing to pay for welfare-friendly meat led to our hypothesis that pet owners in America would be more interested in farm animal welfare and willing to pay a premium for welfare-friendly pet food.

Respondents, overall, were less knowledgeable about farm animal welfare than they thought they were. This is consistent with Pirsich’s findings and other studies that found low consumer knowledge of farm animal welfare (Boogaard *et al.* 2006; Stampa *et al.* 2020). Heng *et al.* (2013) also found their respondents relatively uninformed about egg-laying hen agricultural practices, but also found that 85% were willing to pay a premium to improve welfare attributes for the layers. Similarly, in our findings, lower actual knowledge of farm animal welfare did not imply lower interest or WTP and interest scores varied among demographic categories. Cat owners provided an interesting dynamic, being more knowledgeable than dog owners and with a higher WTP for welfare-friendly pet food, despite a slightly lower interest score.

Overall, both dog and cat owners displayed a positive mean WTP for animal welfare-friendly pet food as defined by our sample images. WTP was largely unpredictable for most demographic variables when looking at pet owners as a whole, with the exceptions of gender and presence of children in the household. Women were more willing to pay for welfare-friendly pet food, while the presence of children in the household decreased WTP. Correlation of WTP with demographic categories was not consistent between dog and cat owners and not clearly linked to household income or education. Cat owners displayed a slightly higher and much more consistent WTP price point for welfare-friendly pet food than dog owners, who were quite diverse in their responses. Younger dog owners displayed the highest stated WTP for welfare-friendly pet food, decreasing as age increased. This heterogeneity in WTP premiums seems consistent with the diversity of the pet food market itself. A wide range of price points and niche market offerings exists even within a single brick-and-mortar or online pet food retailer. The pet food market does not seem to require a ‘one size fits all’ approach.

Despite these positive results indicating that pet owners are willing to pay a premium for pet food that incorporates welfare-friendly ingredients, stated WTP does not always correlate to real life purchasing behaviour (Louviere *et al.* 2000; Lusk and Schroeder 2004). In our findings, stated WTP was not correlated to household income, and younger pet owners claimed the highest WTP. This creates scepticism as to whether these consumers could truly afford the price premium they claimed to be willing to pay or if their responses are based on hypothetical idealism.

Two approaches to converting demonstrated interest in welfare-friendly products include improving consumer knowledge about agricultural practices and reducing the overall cost of welfare-friendly production. Toma *et al.* (2010) and Heng *et al.* (2013) found that providing information to respondents shifted WTP and purchasing behaviour. In contrast, a limited-population study of university-affiliated respondents did not find that providing information only about humane farming practices affected WTP for dairy products (Elbakidze & Nayga Jr 2012). The authors hypothesised that consumers may have too little knowledge of agricultural practices to recognise that the humane care claims differed from conventional practices. Any information provided may need to illustrate how the welfare-friendly products differed from conventional ones to be impactful.

Pirsich *et al.* (2017) argue that utilising specialty pet food as an avenue for welfare-friendly by-products could give producers the opportunity to charge a premium for them, making it more cost-effective, potentially reducing the purchase price of welfare-friendly food products for both humans and pets.

There is debate about whether regulation or voluntary market forces such as labelling schemes and consumer demand are most effective to create widespread improvements in farm animal welfare (for an example related to California’s Proposition 2, see Allender & Richards 2010). Buller (2018) discussed how marketing strategies aimed at the consumer not only meet existing consumer demand, but also shape it. This effect has been seen in influential marketing trends in pet food that are not rooted in nutritional science, such as the ‘grain-free’ trend. Given pet owners’ commitment to feeding their pets what they perceive to be the best, and their increased concern about farm animal welfare, marketing pet food as ‘welfare-friendly’ may be an avenue toward increasing demand for better animal welfare practices that would also trickle up to food for human consumption.

A limitation of our study included the fact that pet owners appeared to self-select for participation in this survey, and so we were unable to compare farm animal welfare knowledge and interest scores with non-pet owners. The introduction to the survey included a statement explaining that participants need not be pet owners to participate, but due to the way that participants search or browse mTurk’s platform, more pet owners may have been drawn to the survey. An opportunity exists for further research in a different setting that would draw more equally from both populations.

### Animal welfare implications

Despite increasing public concern about farm animal welfare, legislative enforcement is yet to be implemented in the US and changes to welfare practices on-farm are driven primarily by consumer demand. Food products advertised as ‘welfare-friendly’ still represent a small segment of the grocery market. The reasons for this are likely multifaceted and include lack of knowledge about current farming practices and economic concerns at the point of sale. Pet food is a robust industry with a strong dependence on marketing trends. Given that pet owners appear to be more interested in farm animal welfare than non-pet owners, and often willing to spend money on their pets that they may not spend on themselves, creating pet foods that utilise welfare-friendly meat products could improve farm animal welfare through a variety of mechanisms. Creating a labelling scheme and providing information about the animal welfare and environmental sustainability practices of the product could raise both consumer awareness and demand as well as making it more economically viable for producers to adopt these practices by allowing them to charge more for their secondary products.

## Conclusion

Pet owners stated an interest in farm animal welfare and a WTP for pet food that contained farm animal-derived ingredients claiming to originate from welfare-friendly practices. These results were largely uncorrelated with household income or education level, indicating that a significant market opportunity may exist for this type of pet food product. Utilising animal-derived ingredients from welfare-friendly farming practices could be an opportunity to meet and increase consumer awareness and demand for improved farm animal welfare and create more economic motivation for producers to adopt improved welfare practices.
